# Elimination of microglia improves cognitive function following cranial irradiation

**DOI:** 10.1038/srep31545

**Published:** 2016-08-12

**Authors:** Munjal M. Acharya, Kim N. Green, Barrett D. Allen, Allison R. Najafi, Amber Syage, Harutyun Minasyan, Mi T. Le, Takumi Kawashita, Erich Giedzinski, Vipan K. Parihar, Brian L. West, Janet E. Baulch, Charles L. Limoli

**Affiliations:** 1 Department of Radiation Oncology, University of California Irvine, CA 92697, USA; 2 Department of Neurobiology and Behavior, University of California Irvine, CA 92697, USA; 3 Plexxikon Inc., Berkeley, California, 94710, USA

## Abstract

Cranial irradiation for the treatment of brain cancer elicits progressive and severe cognitive dysfunction that is associated with significant neuropathology. Radiation injury in the CNS has been linked to persistent microglial activation, and we find upregulation of pro-inflammatory genes even 6 weeks after irradiation. We hypothesize that depletion of microglia in the irradiated brain would have a neuroprotective effect. Adult mice received acute head only irradiation (9 Gy) and were administered a dietary inhibitor (PLX5622) of colony stimulating factor-1 receptor (CSF1R) to deplete microglia post-irradiation. Cohorts of mice maintained on a normal and PLX5662 diet were analyzed for cognitive changes using a battery of behavioral tasks 4–6 weeks later. PLX5622 treatment caused a rapid and near complete elimination of microglia in the brain within 3 days of treatment. Irradiation of animals given a normal diet caused characteristic behavioral deficits designed to test medial pre-frontal cortex (mPFC) and hippocampal learning and memory and caused increased microglial activation. Animals receiving the PLX5622 diet exhibited no radiation-induced cognitive deficits, and exhibited near complete loss of IBA-1 and CD68 positive microglia in the mPFC and hippocampus. Our data demonstrate that elimination of microglia through CSF1R inhibition can ameliorate radiation-induced cognitive deficits in mice.

Microglia are the principal immune cells of the central nervous system (CNS) that respond to injury, infection or disease to eliminate accumulated debris thereby serving a neuroprotective role. Representing ~12% of all CNS cell types, they are ubiquitously spread throughout the brain and have recently been shown to be dependent on colony-stimulating factor 1 receptor (CSF1R) signaling for their survival^1^. Due to its key role in brain development ablation of this gene leads to early death in CSF1R knockout mice^2^,^3^. In the undamaged adult brain, microglia are the main cell type expressing CSF1R and targeted inhibition of this signaling axis leads to a rapid and near complete elimination of microglia^1^,^4^. Interestingly, adult mice devoid of microglia exhibit no overt or persistent abnormalities or adverse effects on cognition, which brings into question their long-term functional role in the intact CNS. Removal of CSF1 inhibition leads to a rapid repopulation of these cells, also with no apparent adverse repercussions^1^,^4^.

Within and outside the CNS, CSF1 signaling plays important immune regulatory roles that can impact cancer therapy. Signaling through CSF1 has been shown to suppress tumor immunity through the recruitment of tumor-infiltrating myeloid cells, and that CSF1R blockade through the use of PLX3397, a related tyrosine kinase inhibitor to PLX5622, could improve immunotherapy in mouse melanoma models^5^,^6^. Similarly, disruption of CSF1R has been shown to impair proliferation *in vitro* and suppress tumor growth using a xenograft model of peripheral nerve sheath tumors^7^. Radiotherapy effectively controls many malignancies, but elicits acute and chronic side effects that are mediated, in part, by persistent inflammatory signatures. This has been exploited in several recent studies showing that the radiotherapeutic response of tumors could be enhanced through CSF1R blockade. Inhibition of CSF1R was found to promote efficient depletion of macrophages and significantly delay tumor regrowth following irradiation^8^. This relationship has been clearly demonstrated in human pancreatic neuroendocrine tumors that have been shown to be dependent on CSF1 signaling for survival and proliferation^9^. PLX3397 also readily crosses the blood brain barrier to deplete CD11b+ myeloid cells and potentiate the response of intracranial tumors to irradiation. Increased efficacy of this treatment has been attributed to preventing irradiation-recruited monocytes from differentiating into immunosuppressive tumor-associated macrophages^10^. Despite this seemingly promising advance in therapeutic approach, an early phase II clinical study of recurrent GBM patients treated with PLX3397 (no irradiation) failed to demonstrate efficacy^11^. However, PLX3397 has shown promising efficacy against tenosynovial giant-cell tumors, with treatment resulting in prolonged regression of tumor volume in most patients in a Phase II trial^12^.

Radiotherapy has been used for decades to forestall the growth of multiple neoplasms, and remains the most effective treatment for brain cancer. Unfortunately, cranial irradiation is associated with significant normal tissue complications, leading to a battery of neurocognitive sequelae that are both progressive and persistent, and adversely impact quality of life for many cancer survivors^13^,^14^. Radiation-induced cognitive dysfunction is a multifaceted disorder caused by elevated oxidative stress, neuroinflammation, decline in neurogenesis^15^,^16^,^17^ and a degradation of neuronal structure and synaptic integrity^18^,^19^,^20^,^21^. These damage signatures are hallmarks of CNS radiation injury that trigger neural circuits to engage a complex remodeling process involving significant structural and synaptic plasticity in the brain^19^,^22^. While many mechanisms influence these dynamic processes, microglia play an active role in reshaping the connective landscape of the brain by selectively pruning dendritic architecture and synapses^23^. However, the degree to which microglial-dependent activities in the irradiated brain are beneficial or harmful to CNS functionality remains unclear. Therefore, based on the growing number of studies targeting CSF1R signaling for therapeutic gain, particularly after radiotherapy, we have investigated the potential benefits of CSF1R blockade after cranial radiotherapy using a selective and brain penetrant inhibitor called PLX5622, which is a sister compound to PLX3397^24^.

## Results

### CSF1R inhibition reduces the number of microglia in the adult brain without affecting cognition

**Figure 1:**
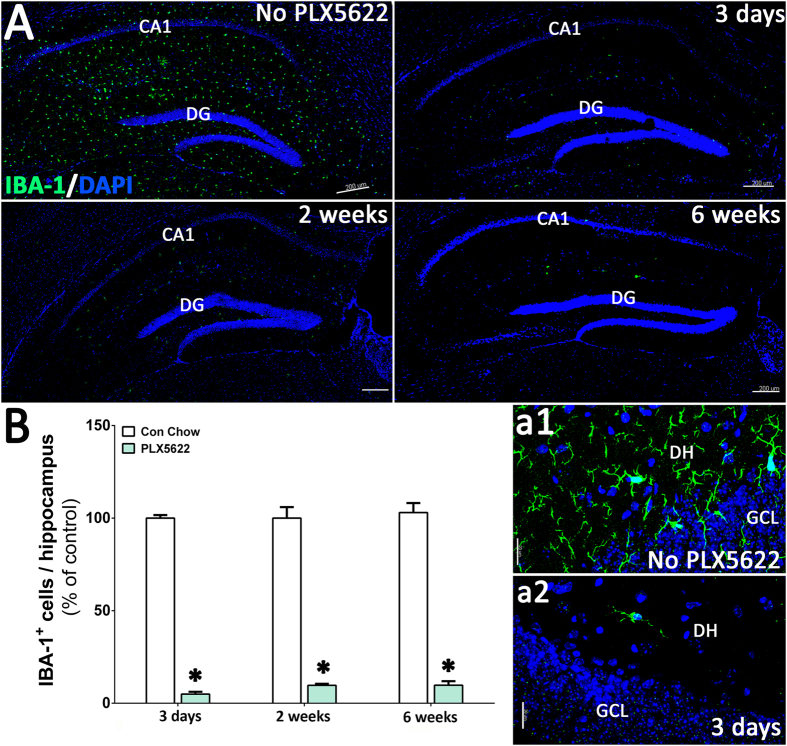
CSF1 receptor (CSF1R) inhibition eliminates microglia from the adult mouse brain. Six month old, male wild type mice were administered control or PLX5622 rodent chow for 3 days, 2 weeks or 4–6 weeks. (**A**) Immunofluorescence staining and laser scanning confocal microcopy was performed on representative brain sections from each group for the microglial marker IBA-1. PLX5622 treatment led to rapid elimination of IBA-1^+^ microglia throughout the brain, with nearly complete elimination after 6 weeks of treatment. (a1,a2) Representative high-resolution (60×) confocal micrographs from the hippocampal dentate hilus (DH) and granule cell layer (GCL) are shown for the control and PLX5622 treated groups for 3 day treatment. (**B**) 3D algorithm-based z stack deconvolution (AutoQuant) and quantification (Imaris) of the number of IBA-1^+^ microglial cell bodies from the hippocampal region show robust decreases in microglial numbers for all the treatment time points. Data are presented as mean ± SEM (*N* = 4 animals/group). *P* values are derived from ANOVA and Bonferroni’s multiple comparisons test. *P < 0.001 compared with control group. Scale bars: 200 μm (**A**) and 20 μm (a1,a2).

**Figure 2:**
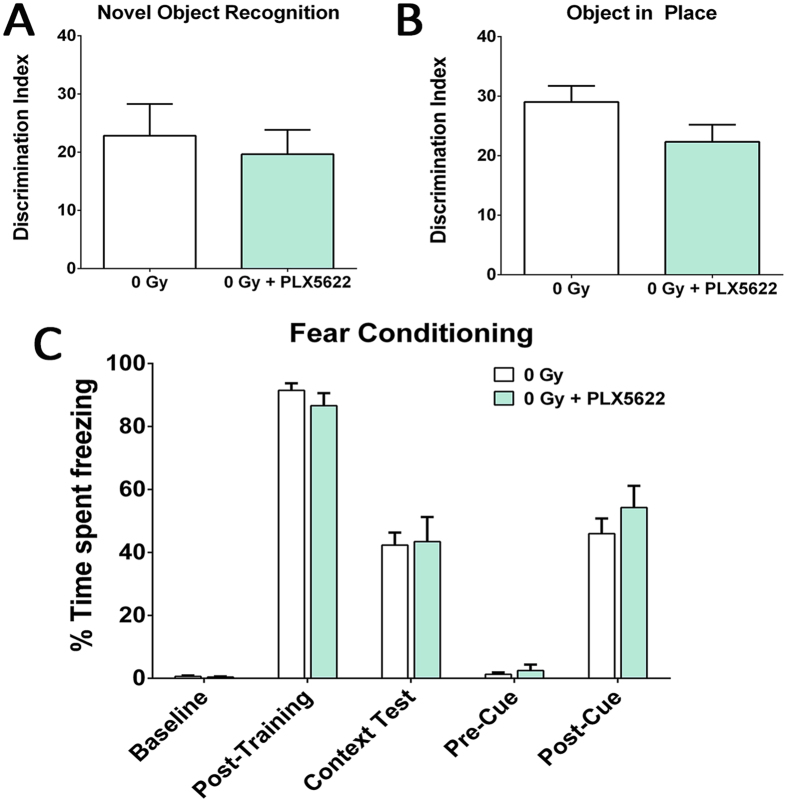
Treatment with CSF1R inhibitor did not affect cognitive function in the intact animals. Unirradiated (0 Gy) adult mice received PLX5622 (1200 mg/kg) or control chow for one month and tested on the hippocampal- and medial pre-frontal cortex (mPFC)-dependent cognitive function tasks (novel object recognition, NOR; object in place, OIP and fear conditioning, FC, **A–C**). The discrimination Index (DI), calculated as ([Novel location exploration time/Total exploration time] − [Familiar location exploration time/Total exploration time]) × 100. PLX5622 treatment did not affect cognitive function as indicated by indistinguishable performance of 0 Gy + PLX5622 and 0 Gy groups. Data are presented as mean ± SEM (*N* = 8–10 mice/group).

For this study we used PLX5622 that specifically inhibits CSF1R tyrosine kinase activity with 50-fold selectivity over 4 related kinases^24^. Six-month old C57Bl/6 wild type mice were treated with PLX5622 (formulated at 1200 mg/kg of chow) for 3 days, 2 weeks or 6 weeks and then microglia were immunostained using the pan-microglial marker, Ionized calcium Binding Adapter molecule (IBA-1, Fig. 1). Laser scanning confocal microscopy (Fig. 1A) through the hippocampal region and quantification (AutoQuant and Imaris, Fig. 1B) revealed that PLX5622 treatment led to significant reductions (95%) in microglial numbers within 3 days. Numbers of microglia remained low (~90%) over the duration of treatment (2 or 6 weeks). These results show that CSF1R-specific inhibition effectively eliminated microglia from the adult brain. Moreover, and validating previous findings^24^, unirradiated mice treated with PLX5622 for 6 weeks showed no adverse effects of CFSR1 blockade on hippocampal- and medial pre-frontal cortex (mPFC)-dependent tasks (novel object recognition, NOR; object in place, OIP; and fear conditioning, FC, Fig. 2). Cognitive function of mice receiving PLX5622 treatment was comparable to mice receiving control diet (Fig. 2).

### CSF1R inhibition ameliorates radiation-induced cognitive dysfunction

**Figure 3:**
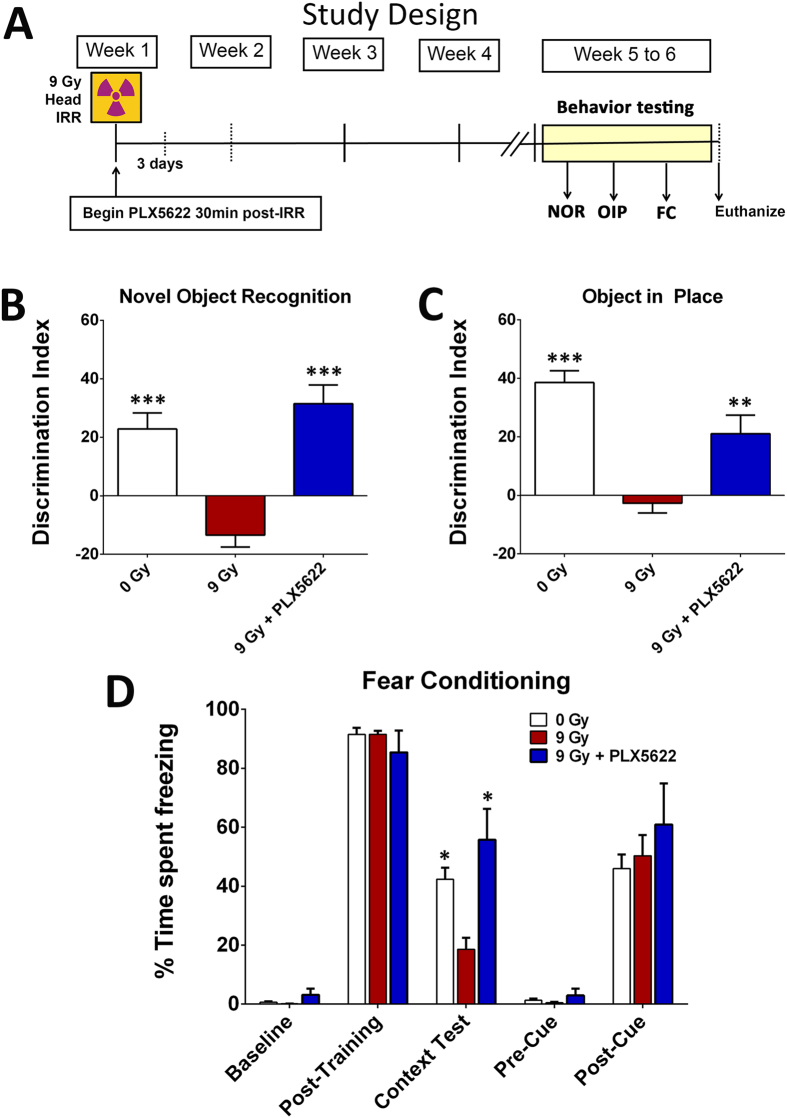
CSF1R inhibition prevents cranial radiation-induced cognitive dysfunction. (**A**) Schematic of study design: 6 month old wild type male mice were irradiated (0 or 9 Gy, head only) and administered CSF1R inhibitor PLX5622 in rodent chow after irradiation (IRR) and continued on diet till the end of study. Animals that received control chow served as vehicle group. A small cohort of mice was euthanized at 3 days and 2 weeks of PLX5622 treatments for assessment of microglial elimination. One month post-IRR and PLX5622 treatment (week 5 to 6), mice were tested on spatial and episodic memory retention using the novel object recognition (NOR) and object in place (OIP) tasks followed by fear conditioning (FC) task. **(B**–**D)** The tendency to explore novel location(s) was derived from the Discrimination Index (DI), calculated as ([Novel location exploration time/Total exploration time] − [Familiar location exploration time/Total exploration time]) × 100. Cranial irradiation (9 Gy) show significant behavioral deficits on the NOR and OIP tasks compared to controls (0 Gy) as indicated by impaired preference to novel object (**B**) or place (**C**). Irradiated animals receiving CSF1R inhibitor (9 Gy + PLX5622) show significant preference for the novelty when compared with irradiated (9 Gy) animals receiving vehicle. **(D)** CSF1R inhibitor improves behavior on the hippocampal-dependent contextual fear-conditioning task. The baseline freezing levels were comparable among groups, and all groups showed elevated freezing behavior following a series of 5 tone-shock pairings (post-training bars). The context test was administered 24 hour later, and irradiated mice (9 Gy) showed significantly decreased freezing compared to controls (0 Gy). Irradiated animals receiving PLX5622 diet (9 Gy + PLX5622) showed a significant elevation in freezing behavior that was indistinguishable from the 0 Gy group. After the initial training phase (48 hours), the context was changed that resulted in a considerable reduction in freezing behavior (Pre-Cue bar**s**) that was restored following the tone sound (Post-Cue test bars), indicating intact amygdala function in all groups. Data are presented as mean ± SEM (*N* = 8–10 mice/group). *P* values are derived from ANOVA and Bonferroni’s multiple comparisons test. ***P < 0.001; **P < 0.01, *P < 0.05 compared with the 9 Gy group.

The irradiated brain exhibits persistent and cyclical cascades of inflammation that impact cognitive function^18^,^25^,^26^,^27^. Despite the importance of inflammatory processes in several neurodegenerative conditions, little or nothing is known about the impact of CSF1R inhibition and microglia depletion on cognitive function following cranial irradiation. In this study, adult male mice received 0 or 9 Gy head-only irradiation and were then immediately placed on a diet with or without PLX5622 for 1-month at which time animals underwent cognitive testing (Fig. 3A).

#### Novel Object Recognition (NOR)

One month post-treatment, mice were habituated in an open field arena and then tested on the NOR task (Fig. 3B). Impairments in the perirhinal cortex and hippocampus manifest as an inability to discriminate a novel from a familiar object in the NOR task^28^,^29^. The total exploration times for both objects were not different between any experimental groups. In the test phase, a significant overall group difference was found between the cohorts for the discrimination index, DI (F_(3,34)_ = 19.49, *P* = 0.0001). After a 5 minute retention interval between the familiarization and test phases, unirradiated mice (0 Gy) showed a preference for the novel object. While irradiated mice (9 Gy) showed a drastic reduction in the preference to explore novelty compared to control mice (*P* = 0.001), irradiated mice administered the PLX5622 chow (9 Gy + PLX5622) showed significantly improved performance on the NOR task compared to the 9 Gy group (*P* = 0.001). The DIs for 0 Gy and 9 Gy + PLX5622 groups were statistically indistinguishable. These data demonstrate that microglial depletion through CSF1R inhibition improves novel object exploration behavior in irradiated animals.

#### Object in Place (OIP)

After completion of NOR testing, mice were habituated and tested on the OIP task (Fig. 3C). Performance on the OIP task is also dependent on the intact hippocampal function in addition to the prefrontal and perirhinal cortices^28^,^29^. Mice with intact cortical function exhibit preference towards objects that have been switched to a novel location. As in the NOR task, the total exploration times for both objects were not different between any experimental groups. For the test phase, a significant overall group difference was found between the cohorts for the DI (F_(2,36)_ = 28.32, *P* = 0.0001). As expected, unirradiated mice (0 Gy) showed a strong preference for the object placed at a novel location while irradiated mice (9 Gy) showed little preference to explore any of the objects and exhibited significant decrements compared to 0 Gy controls (*P* = 0.001). Irradiated mice treated with PLX5622 (9 Gy + PLX5622) showed a strong preference for the objects placed at a novel location that was significantly different (*P* = 0.01) from the 9 Gy group. The 0 Gy and 9 Gy + PLX5622 groups were again statistically indistinguishable. These data further demonstrate that microglial elimination is associated with significant improvements in spatial exploration on the OIP task in the irradiated mice.

#### Fear Conditioning (FC)

The fear-conditioning paradigm included training, context and cue testing phases administered over 3 days (Fig. 3D). Following the tone-shock training, animals showed significant freezing (Post-training columns). Group means and 95% CIs for the post-training freezing (percent) were as follows: 0 Gy (mean = 91.47, 95% CI = 85.67–97.26); 9 Gy (mean = 91.53, 95% CI = 88.50–94.55); 9 Gy + PLX5622 (mean = 85.40; 95% CI = 64.91–105.9). Since all groups demonstrated significant increases in freezing behavior after the tone-shock pairings during the post-training phase, cranial irradiation did not impair motor or sensory function. Twenty-four hours after tone-shock training, mice were administered the context phase of testing, where animals with intact hippocampal function show increased freezing behavior. Repeated measure (RM) ANOVA for the context phase revealed significant differences between the 9 Gy and 0 Gy groups (*P* = 0.05) and between the 9 Gy + PLX5622 and 9 Gy groups (*P* = 0.05), indicating that significant radiation-induced impairments in hippocampal memory could be restored by PLX5622 treatment. Groups did not differ significantly in the freezing behavior across baseline, post-training, pre-cue and post-cue phases (Fig. 3D), indicating a selective deficit on the hippocampal-dependent contextual memory phase of the task^30^,^31^, in the absence of amygdala-dependent deficits in cued memory acquisition of the tone-shock pairing. These data corroborate our prior cognitive testing trends and demonstrate that radiation-induced deficits in a third cognitive task could be ameliorated by CSF1R blockade and microglial depletion.

### CSF1R blockade reduces microglial populations in the unirradiated and irradiated hippocampus

**Figure 4:**
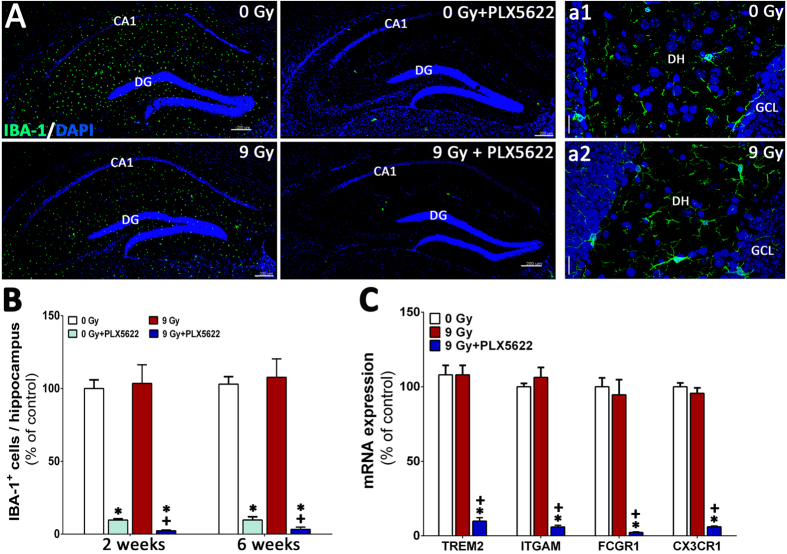
CSF1R inhibition eliminates IBA-1^+^ microglia from the unirradiated and irradiated hippocampus. (**A**) Immunofluorescence staining and laser scanning confocal microscopy demonstrates that PLX5622 treatment for 6 weeks eliminates ~90% of IBA-1^+^ microglia from the control (0 Gy + PLX5622) and irradiated (9 Gy + PLX5622) brains. (a1,a2) Representative high-resolution (60×) confocal micrographs from the hippocampal dentate hilus (DH) and granule cell layer (GCL) are shown for the 0 and 9 Gy mice that received control chow. (**B**) 3D algorithm-based quantification (Autoquant and Imaris) of IBA-1^+^ microglia show almost complete elimination (90–96%) of microglia in the control and irradiated brains of animals treated with PLX5622 (0 Gy + PLX5622 and 9 Gy + PLX5622) at 2 week and 6 week post-treatments. (**C**) Gene expression analysis of microglial markers from whole brains derived from a separate cohort of irradiated animals (0 and 9 Gy) received 1 week of PLX5622 treatment at 4 week post-irradiation show 80–90% reduction in mRNA levels after treatment with PLX5622. Data are presented as mean ± SEM (*N* = 4 mice/group). *P* values are derived from ANOVA and Bonferroni’s multiple comparisons test. *P < 0.001 compared with 0 Gy group and ^+^*P* < 0.001 compared with the 9 Gy group. Scale bars: 200 μm (**A**) and 20 μm (a1,a2).

To confirm that the beneficial neurocognitive effects of CSF1R inhibition in the irradiated brain were in fact associated with microglial elimination, we quantified the number of IBA-1^+^ microglia (Fig. 3). Hippocampal sections from four groups of animals (0 Gy ± PLX5622 and 9 Gy ± PLX5622) were immunostained and scanned using confocal microscopy (Fig. 4A,a1,a2). 3D algorithm based quantification (AutoQuant and Imaris) of confocal z stacks indicated that PLX5622 treatment led to robust reductions (*P* = 0.001) in the number of IBA-1^+^ microglia at 2 and 6 week post-treatment in the unirradiated control animals where more than 90% of IBA-1^+^ microglia were eliminated at both time points (Fig. 4B). Cranial irradiation alone led to swelling in IBA-1^+^ cell bodies (percentage of control, 130.62 ± 2.12, mean ± SEM, n = 3) but did not change the number of IBA-1^+^ microglia from control levels. However, PLX5622 treatment did cause significant reductions (>96%, *P* = 0.001) in the number of IBA-1^+^ cells in the irradiated hippocampus at 2 and 6 weeks after irradiation. These data demonstrate a correlation between the presences of microglia in the irradiated brain and adverse effects on cognition.

### Inhibition of CSF1R attenuates microglial activation in the irradiated hippocampus

**Figure 5:**
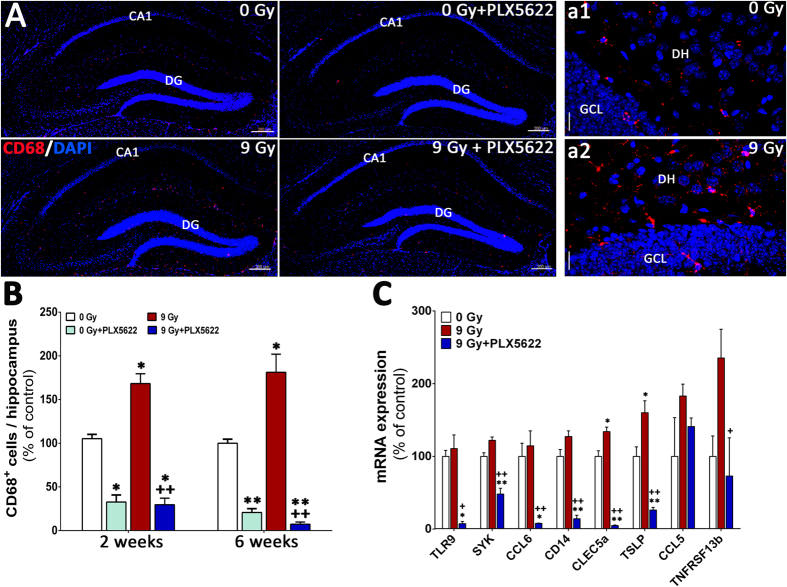
CSF1R inhibition reduces CD68^+^ activated microglia from the irradiated hippocampus. (**A**) Immunofluorescence staining show that PLX5622 treatment for 6 weeks reduces ~70% of CD68^+^ activated microglia from the control (0 Gy + PLX5622) and irradiated (9 Gy + PLX5622) brains. (a1,a2) Representative high-resolution (60×) z stacks show ramified microglial morphology in the irradiated hippocampal dentate hilus (DH) and granule cell layer (GCL) compared to 0 Gy mice that received control chow. (**B**) Quantification (Autoquant and Imaris) of CD68^+^ activated microglia indicated an 80–90% reduction in the control and irradiated brains receiving PLX5622 (0 Gy + PLX5622 and 9 Gy + PLX5622) at 2 week and 6 week time points. (**C**) Analysis of pro-inflammatory markers from whole brains derived from irradiated mice (0 and 9 Gy) treated with PLX5622 for 1 week at 4 week post-irradiation show radiation-induced elevation in gene expression that was reduced significantly by PLX5622 treatment. Data are presented as mean ± SEM (*N* = 4 mice/group). *P* values are derived from ANOVA and Bonferroni’s multiple comparisons test. *P < 0.01; **P < 0.001 compared with 0 Gy group and ^+^*P* < 0.01; ^++^*P* < 0.01compared with 9 Gy group. Scale bars: 200 μm (**A**) and 20 μm (a1,a2).

Radiation-induced microglial activation plays a contributory, if not causal, role in many aspects of cognitive dysfunction^18^,^26^,^27^. To determine the impact of CSF1R inhibition on activated microglia in the irradiated brain, animals from each cohort (0 Gy ± PLX5622 and 9 Gy ± PLX5622) were immunostained for the activated microglial marker (CD68) and quantified using confocal microscopy and Imaris (Fig. 5). Compared to controls, irradiation led to a significant increase (1.5–2 fold) in the number of CD68^+^ activated microglia with ramified morphology in the hippocampus at 2 and 6 week post-irradiation (Fig. 5A,a1,a2). Conversely, animals treated with PLX5622 had significantly reduced (~70%) numbers of CD68^+^ cells compared to controls at each time point (Fig. 5B). Elimination of activated microglia via PLX5622 treatment was more pronounced the irradiated brain, where 70–95% reductions in the number of CD68^+^ cells were found at 2 or 6 week post-irradiation. A second cohort of mice were subjected to 0 or 9 Gy irradiation and brains extracted 6 weeks later. An additional group was subjected to 9 Gy and then 4 weeks later treated for 1 week with PLX5622. Gene expression profiling of pro-inflammatory markers (TLR9, SYK, CCL6, CD14, CLECL5a, TSLP, CCL5, TNFRSF13b) showed radiation-induced elevation and PLX5622 treatment-mediated reduction in the irradiated brains (Fig. 5C). Additionally, expression analysis of genes associated with microglial function (TREM2, ITGAM, FCGR1 and CX3CR1) demonstrated markedly reduced expression in PLX5622 treated mice, consistent with microglial elimination (Fig. 4C).

### Microglial elimination in the irradiated pre-frontal cortex

**Figure 6:**
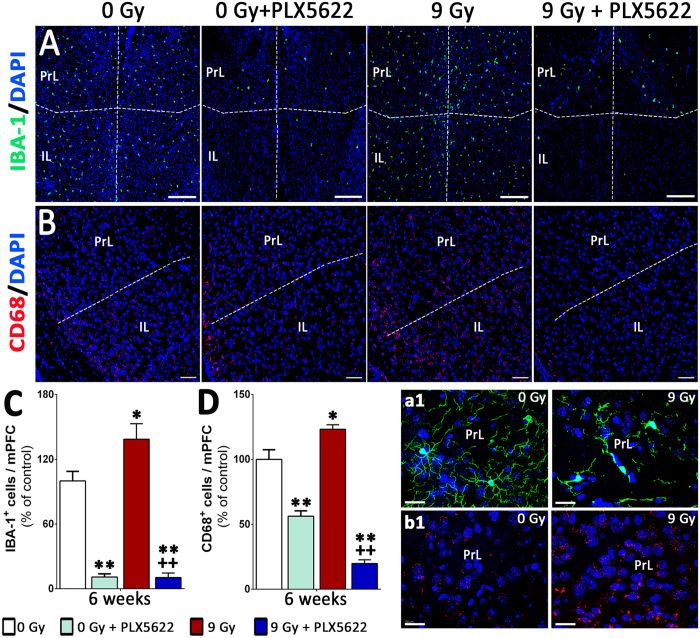
Microglial depletion in the medial pre-frontal cortex by CSF1R inhibition. (**A,B**) Immunofluorescence staining and laser scanning confocal microscopy for the IBA-1^+^ and CD68^+^ cells show that cranial irradiation lead to significant elevation in microglial number (40% and 25% respectively) in the pre-limbic (PrL) and infra-limbic (IL) cortices of the medial pre-frontal cortex (mPFC). (a1,b1) Representative high-resolution (60×) z stacks showed characteristic activated microglial morphology (IBA-1, a1 and CD68, b1) in the irradiated PrL compared to 0 Gy group. (**C,D**) Treatment with PLX5622 for 6 weeks eliminates 80–90% of IBA-1^+^ and CD68^+^ microglia from the control (0 Gy + PLX5622) and irradiated (9 Gy + PLX5622) mPFC. Data are presented as mean ± SEM (*N* = 4 mice/group). *P* values are derived from ANOVA and Bonferroni’s multiple comparisons test. *P < 0.05; **P < 0.001 compared with 0 Gy group and ^+^*P* < 0.05; ^++^*P* < 0.01compared with 9 Gy group. Scale bars: 200 μm (**A**), 100 μm (**B**) and 50 μm (a1,b1).

Significant decline in performance on the spontaneous exploration, episodic memory tasks (NOR, OIP, Fig. 3B,C) indicate the sensitivity of the hippocampus and pre-frontal cortex to cranial irradiation. To determine the impact of radiation and CSF1R inhibitor treatments, the microglial profile (IBA-1 and CD68) of the medial pre-frontal cortex (mPFC) was evaluated at 6 weeks post-treatment (Fig. 6). Exposure to cranial irradiation significantly elevated numbers of IBA-1 and CD68 positive microglia (40% and 25% respectively, Fig. 6A,B) within the pre-limbic (PrL) and infra-limbic (IL) cortices of the mPFC. Microglia in the irradiated mPFC showed characteristic activation morphology with larger cell bodies (Fig. 6a1) and increased CD68 expression (Fig. 6b1). Treatment with PLX5622 reduced the numbers of IBA-1^+^ and CD68^+^ cells (80–90% reduction, Fig. 6C,D) in the irradiated mPFC. This data illustrate the global impact of systemic CSF1R inhibitor treatment on the microglial profile of irradiated brain.

## Discussion

Current studies leverage our past findings demonstrating that prolonged administration of a CSF1R inhibitor can eliminate nearly all microglia from the adult brain^1^,^24^. Importantly, in normal adult animals devoid of microglia, adverse neurocognitive sequelae were not found and basal motor skills and cognitive functions were intact. While these studies might suggest microglia are dispensable for longer-term neurocognitive health, their known roles in the eradication of cellular debris suggest their true value may come during the ageing process or during recovery from CNS injury, insult or disease. Here we critically tested their functional importance in a well characterized rodent model of radiation injury, where acute exposure to ionizing radiation has been shown to elicit a range of cognitive deficits and associated neuropathology^18^,^19^.

Here, we used the highly specific and brain penetrant CSF1R inhibitor PLX5622^24^. This inhibitor eliminates >95% of microglia from throughout the CNS within 3 days, and has also been shown to eliminate chronically activated microglia from a mouse model of Alzheimer’s disease^32^. In addition, its effects on peripheral circulating myeloid cells have been characterized and shown to have no impact^33^, highlighting the unique dependence of microglia on CSF1R signaling. In accordance with these prior studies, we demonstrated that relatively brief supplementation with PLX5622 caused a rapid depopulation of IBA-1 positive microglia, an effect that was maintained throughout dietary administration. To determine how CSF1R inhibition impacted cognition, animals were subjected to sequential spontaneous exploration tasks (NOR, OIP) followed by a fear-conditioning behavioral paradigm. In this study animals were tested for behavior 4–6 weeks after irradiation, which reflects the delayed onset of cognitive impairments after exposure to cranial irradiation in combination with PLX5622 treatment. Control animals provided PLX5622 showed no neurocognitive abnormalities on any of the behavioral tasks, corroborating past data showing the absence of adverse behavioral complications associated with short (2–6 weeks) or long-term (2–3 months) administration of different CSF1R inhibitors^1^,^4^,^24^,^34^. Given this backdrop, we determined the consequences of microglial elimination in the irradiated brain. As previously observed, acute radiation exposure caused significant decrements in the preference for novelty, as the ability of animals to discriminate new objects or locations was impaired on each of the spontaneous open field tasks. Deficits were also found on the contextual version of a fear-conditioning task, suggesting that neurocognitive sequelae were associated with hippocampal- and cortical-based learning and memory rather than altered locomotion. In each instance however, radiation-induced deficits in all behavioral tasks were significantly ameliorated by microglial depletion, suggesting that disruption of CSF1R signaling had positive neurocognitive benefits over the duration of these studies.

To more conclusively determine how microglial levels correlated with cognitive changes after irradiation, the total yields of IBA-1 positive cells were determined. Irradiation was not found to cause major changes in the yield of IBA-1 positive cells, similar to our past findings with a lipopolysaccharide challenge^1^. Also consistent with past findings, control mice given the PLX5622 exhibited marked reductions in IBA-1 positive microglia with or without irradiation. Immunohistochemical findings were corroborated by RNA analyses, where PLX5622 treatment initiated after irradiation induced significant and dramatic reductions in the message levels of microglial function markers (TREM2, ITGAM, FCGR1 and CX3CR1)^1^. Importantly, CSF1R inhibition was equally effective at reducing IBA-1 positive cells in both control and irradiated cohorts, suggesting that the irradiated microenvironment was persistently responsive to CSF1R blockade. Qualitatively similar results were found when the levels of CD68 positive activated microglia were assessed in the hippocampus. However in this case, irradiation was found to increase the level of microglia expressing the pro-inflammatory marker CD68, similar to past findings showing elevated ED1 positive activated microglia in irradiated rats^18^,^21^. These findings were also corroborated by additional analyses that showed radiation-induced increases in the relative mRNA levels for pro-inflammatory genes were reduced by CSFR1 blockade after irradiation. Collectively, these data provide convincing evidence that basal and radiation-induced increases in activated microglia were effectively ablated by PLX5622 mediated inhibition of CSF1R.

The results of this study demonstrate the positive neurocognitive benefits of microglial elimination in the irradiated brain. The fact that CSF1R expression is specific for microglia and induces relatively undetectable side effects makes this strategy an attractive therapeutic intervention for ameliorating the adverse effects of cranial irradiation on cognition. Moreover, our past studies showed that withdrawal of CSF1R inhibitor treatment led to a nearly complete repopulation of microglia within 3 to 7 days, indicating the potential safe clinical application of this strategy. Whether CSF1R inhibition affects cognitive function at long-term post-irradiation time points, or whether this strategy is equally effective after clinically relevant fractionated irradiation paradigms remains to be determined. It should be noted that the timing of microglial elimination is also likely to be important for beneficial vs. detrimental effects, as microglia have protective as well as harmful effects on the brain in an injury setting. For example, we found that elimination of microglia followed by a neuronal insult exacerbated the injury, while elimination after the neuronal injury improved recovery^34^. Another recent study showed that elimination of microglia followed by a stroke dramatically increased the infarct size^35^. Overall, recent reports indicating that CSF1R blockade may be useful for enhancing tumor control through the suppression of tumor immunity suggest that strategies pursuing the inhibition of CSF1R may operate through multiple mechanisms for improving clinical outcomes in cancer patients.

## Materials and Methods

### Animals, irradiation and CSF1R inhibitor treatment

All animal procedures described in this study are in accordance with NIH guidelines and approved by the University of California Institutional Animal Care and Use Committee. Six month old male wild type mice (C57Bl/6J, Jackson) were maintained in sterile housing conditions (20 °C ± 1 °C; 70% ± 10% humidity; 12 h:12 h light and dark cycle) and had free access to standard rodent chow and water. The mice were divided into 4 experimental groups (*n* = 8–12 mice per group): unirradiated receiving control chow (0 Gy or Control), unirradiated receiving CSF1R inhibitor (0 Gy + PLX5622), head-only irradiation receiving control chow (9 Gy) and head only irradiation receiving CSF1R inhibitor (9 Gy + PLX5622). For cranial irradiation, mice were anesthetized (5% induction and 2% maintenance isoflurane, vol/vol), placed ventrally on the treatment table (XRAD 320 irradiator, Precision X-ray, North Branford, CT) without restraint, and positioned under a collimated (1.0 cm^2^ diameter) beam for head-only irradiation such that cerebellum, eyes and rest of the body were shielded from the radiation exposure. The irradiator was equipped with a hardening filter (0.75mm Sn + 0.25mm Cu + 1.5mm Al; HVL = 3.7mm Cu, half value layer) to eliminate low energy X-rays to minimize skin damage. X-irradiation was delivered at the dose rate of 1.10 Gy/min. After irradiation mice were provided control or PLX5622 chow. CSF1R inhibitor, PLX5622, was provided by Plexxikon (Berkeley, CA) and formulated in AIN-76A standard chow by Research Diets (New Brunswick, NJ) at a dose of 1200 mg/kg. Control mice received AIN-76A chow without PLX5622. All mice were maintained on their respective PLX5622 or control diet from immediately post-irradiation throughout the duration of all studies.

### Cognitive testing

To determine the effects of CSF1R inhibition on cognitive function after irradiation, mice were subjected to behavioral testing 1 month after irradiation. Testing was conducted over 2 weeks and included two open field, spontaneous exploration tasks (novel object recognition (NOR) and object in place (OIP)) followed by a fear conditioning (FC) task. Behavioral testing followed our previously described protocols^36^,^37^. Data analysis was conducted independently and blind and presented as the average of all trials scored for each task. Animals were first administered the NOR task followed by the OIP task. Time spent exploring both familiar and novel place or object was counted when the nose of the mouse was within 1 cm and pointed in the direction of the object. Mice did not show object climbing or neophobic behavior. NOR and OIP data are presented as a discrimination index (DI) and calculated as ([Novel location exploration time/Total exploration time] − [Familiar location exploration time/Total exploration time]) × 100. A positive index indicates that a mouse spent more time exploring novelty (*i.e.* switched objects or locations), while a negative score indicates little or no preference for exploring novelty. The FC task was administered in three sequential phases over three days including a training phase, a context test and a cue test as described previously^36^,^37^.

### Immunohistochemistry, confocal microscopy, and quantification

At select times (2 days, 2 week or 6 week), animals were deeply anesthetized with isoflurane and euthanized with saline with heparin (10 U/ml, Sigma-Aldrich) followed by 4% paraformaldehyde (intracardiac perfusion). Brains were cryoprotected (30% sucrose) and sectioned coronally (30 μm thick) using a cryostat (Leica Microsystems, Germany). For the immunofluorescence analysis of microglia, the following primary and secondary antibodies were used: rabbit anti-IBA-1 (1:500, Wako), rat anti-mouse CD68 (1:500, AbD Serotec), donkey anti-rabbit or anti-mouse conjugated with Alexa Fluor 488 or 594 (Life Technologies/Invitrogen) and DAPI nuclear counterstain (Sigma-Aldrich). Representative sections (3–4 sections per animal, 4 animals per group) through the middle of the hippocampus were selected and immunofluorescence staining followed procedures described in detail previously^1^,^18^,^21^. IBA-1 positive cells were visualized under fluorescence as green, CD68 as red and nucleus as blue fluorescence.

Immunofluorescent sections were imaged using Nikon Eclipse Ti C2 microscope to obtain 20 to 30 z stacks (1024 × 1024 pixels, 1 μm each) using 10 and 60× PlanApo oil-immersion lens (Nikon). For quantification of IBA-1^+^ and CD68^+^ cells, 3D deconvolution and reconstruction was carried out using the AutoQuantX3 algorithm (MediaCybernetics). Deconvolution combined with 3D reconstruction yields higher spatial resolution images for the immunofluorescent cell bodies and stellae. Quantification was facilitated using Imaris spot tool (v8.0, Bit Plane Inc., Switzerland) that detect immunostained puncta within 3D deconvoluted image stacks based on a predefined diameter and red/green channel intensity threshold. IBA-1 and CD68 data are expressed as mean immunoreactivity (percentage) relative to unirradiated (0 Gy) controls.

### Gene expression analysis

Adult wild-type mice on control diet were head-irradiated (0 or 9 Gy) as described above. Four weeks later, one cohort of mice was treated with PLX5622 for 7 days to eliminate microglia (n = 4), resulting in three groups (0 Gy + Control, 9 Gy + Control, and 9 Gy + PLX5622). Mice were sacrificed 5- (9 Gy + PLX5622) or 6 weeks post-irradiation (0 Gy + Control, 9 Gy + PLX5622). Total mRNA was extracted from half-brains using an RNA Plus Universal Mini Kit (Qiagen), and was hybridized and multiplexed with NanoString probes, according to the manufacturer’s instructions. Microglial function (TREM2, ITGAM, FCGR1 and CX3CR1) and pro-inflammatory (TLR9, SYK, CCL6, CD14, CLECL5a, TSLP, CCL5, TNFRSF13b) genes were selected for analysis using the NanoString nCounterTM mouse immunology panel (NanoString). Counts for target genes were normalized to house-keeping genes (EEF1G, G6PDX, HPRT, POLR1B, POLR2A, PPIA, RPL19, SDHA and TBP) to account for variability in the RNA content. Background signal was calculated as a mean value of the negative hybridization control probes. Gene expression values were presented as percentage of control (0 Gy) group.

### Statistical analysis

Statistical analyses were carried out using GraphPad Prism (v6) software. One-way ANOVA were used to assess significance between control and irradiated groups receiving either control or PLX5622 chow. When overall group effects were found to be statistically significant, a Bonferroni’s multiple comparisons test was used to compare the 9 Gy with individual experimental groups. For analysis of fear conditioning data, repeated measures two-way ANOVA were performed. All analyses considered a value of *P* ≤ 0.05 to be statistically significant.

## Additional Information

**How to cite this article**: Acharya, M. M. *et al*. Elimination of microglia improves cognitive function following cranial irradiation. *Sci. Rep.* **6**, 31545; doi: 10.1038/srep31545 (2016).
